# Supplementary material for: Complexome profiling of the Chlamydomonas psb28 mutant reveals TEF5 as an early PSII assembly factor
Source: Plant Cell. 2025 Mar 17;37(6):koaf055. doi: 10.1093/plcell/koaf055 (PMC12164590; doi:10.1093/plcell/koaf055)
Supplement: koaf055_Supplementary_Data [file koaf055_supplementary_data.zip › psb28_interactive.zip!/plots/intensity/qualityPlots/peptides/TERC_Cre17.g712400.t1.2.html]

### Cre17.g712400.t1.2

Trivial name: TERC  
SeqCoverage [median]: 9.70  
Mass: 38.72 kDa  
  
 

| Sequence | MissedCleavage | Mass [Da] | wtIntensity | muIntensity | Score |
| --- | --- | --- | --- | --- | --- |
| ELQYISTTLPR | 0 | 1319.71 | 3614.87 | 11474.50 | 152.54 |
| QAEQERELQYISTTLPR | 1 | 2061.05 | 1630.17 | 3220.02 | 44.73 |
| VTDKYDGTNFFTTVNGNR | 1 | 2047.96 | 0.00 | 3085.37 | 60.95 |
|  |  |  | 5245.0 | 17779.9 |  |
